# Perfect Crystals: microgravity capillary counterdiffusion crystallization of human manganese superoxide dismutase for neutron crystallography

**DOI:** 10.1038/s41526-023-00288-x

**Published:** 2023-06-03

**Authors:** William E. Lutz, Jahaun Azadmanesh, Jeffrey J. Lovelace, Carol Kolar, Leighton Coates, Kevin L. Weiss, Gloria E. O. Borgstahl

**Affiliations:** 1 Eppley Institute for Research in Cancer and Allied Diseases, 986805 Nebraska Medical Center, Omaha, NE 68198-6805 USA; 2 Second Target Station, Oak Ridge National Laboratory, 1 Bethel Valley Road, Oak Ridge, TN 37831 USA; 3 Neutron Scattering Division, Oak Ridge National Laboratory, 1 Bethel Valley Road, Oak Ridge, TN 37831 USA; 4 Fred and Pamela Buffet Cancer Center, University of Nebraska Medical Center, Omaha, NE USA

**Keywords:** Structural biology, Biochemistry

## Abstract

The NASA mission *Perfect Crystals* used the microgravity environment on the International Space Station (ISS) to grow crystals of human manganese superoxide dismutase (MnSOD)—an oxidoreductase critical for mitochondrial vitality and human health. The mission’s overarching aim is to perform neutron protein crystallography (NPC) on MnSOD to directly visualize proton positions and derive a chemical understanding of the concerted proton electron transfers performed by the enzyme. Large crystals that are perfect enough to diffract neutrons to sufficient resolution are essential for NPC. This combination, large and perfect, is hard to achieve on Earth due to gravity-induced convective mixing. Capillary counterdiffusion methods were developed that provided a gradient of conditions for crystal growth along with a built-in time delay that prevented premature crystallization before stowage on the ISS. Here, we report a highly successful and versatile crystallization system to grow a plethora of crystals for high-resolution NPC.

## Introduction

Human manganese superoxide dismutase (MnSOD) is an oxidoreductase found in the mitochondrial matrix and is responsible for catalyzing the disproportionation of superoxide into molecular oxygen and hydrogen peroxide using a systematic and efficient array of proton transfers^1^. Despite numerous experiments and X-ray crystal structures, the exact chemistry behind MnSOD function is unclear as the step-by-step proton transfers resulting in the elimination of superoxide are not known. An atomic understanding of MnSOD catalysis requires direct proton visualization, which can be achieved by neutron crystallography^2,3^. The crystals of MnSOD grown in microgravity on the International Space Station (ISS) have enabled the collection of high-resolution neutron diffraction^4^ and will make possible the determination of several neutron structures.

Due to the large unit-cell dimensions of MnSOD crystals, the Macromolecular Neutron Diffractometer (MaNDi) at the Oak Ridge National Laboratory (ORNL) Spallation Neutron Source (SNS) is being actively employed for neutron crystallography diffraction data collection using time-of-flight Laue techniques^5^. With these data, the positions of hydrogens within protein crystals can be determined. For neutrons, the coherent scattering lengths of hydrogen (H) and deuterium (D) are of similar magnitudes to those of other biologically important atoms, e.g., carbon (C), nitrogen (N), and oxygen (O); so they can all be detected simultaneously for the entire enzyme. However, the neutron scattering length of H is negative, while C, N, and O are positive, meaning the presence of hydrogen leads to signal cancellation effects. Fortunately, D has a positive scattering length, and thus the H problem can be circumvented through protein perdeuteration^6^. Though the SNS is one of the world’s highest flux pulsed neutron beams, the flux of the beam is still much lower than the flux of some rotating anode and synchrotron X-ray crystallography sources^7^. This low flux necessitates crystals with volumes 0.1–1.0 mm^3^ to amplify the signal and diffract neutrons to high resolution. Such large-volume, neutron-diffraction quality crystals can be difficult to grow on Earth.

**Fig. 1:**
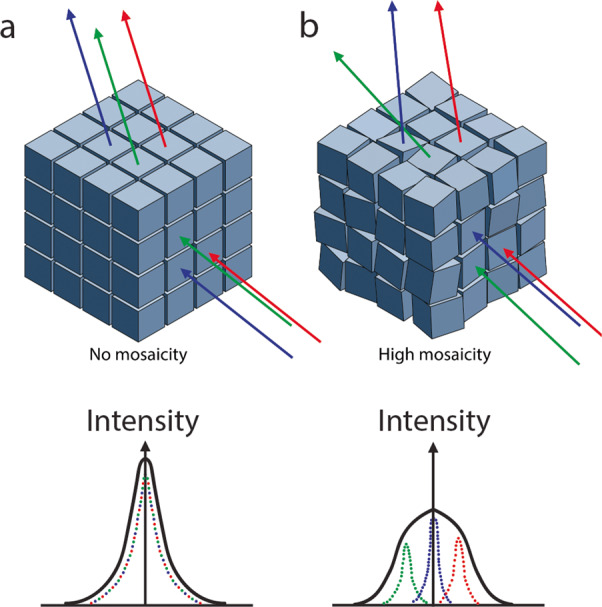
Schematic representation of the effect of crystal mosaicity on diffraction. **a** A perfect crystal with no mosaicity would yield high-quality diffraction data (below). Here the hypothetical mosaic blocks are perfectly aligned (green, blue, and red) and diffract together to form a sharp diffraction reflection with a high signal-to-noise ratio. **b** A crystal with defects would have high mosaicity that would yield data of lesser quality. Here, the hypothetical three mosaic blocks diffract at different angles and, when combined, result in a broadened diffraction reflection with a lower signal-to-noise ratio. Please note that the mosaic domains are not necessarily uniform or cuboid.

Most crystals contain growth defects to a certain extent. These imperfections can be considered small perfect crystals that are randomly misaligned in the context of the surrounding crystal environment, resulting in a “mosaic” of crystals (Fig. 1)^8^. Additionally, the individual protein molecules incorporated at the crystal’s growth interface can misalign. Increased crystal size can be correlated to the increased inclusion of misaligned microcrystals as well as randomly misincorporated protein molecules. Such mosaicity can decrease the quality of neutron and X-ray diffraction data by widening or smearing diffraction peak intensities, and thus crystallographers aim to minimize it (Fig. 1)^9^. To grow crystals that are both large volume and perfect is a difficult task but one that can be achieved with microgravity-crystal growth^10–12^. In particular, early experiments dramatically improved the growth of MnSOD crystals to large volume^13^. Counterdiffusion methods for crystal growth allow the screening of a gradient of crystallization conditions in each experiment^14^ and have been coupled with the microgravity environment to grow large-volume crystals^15^. In the current work, capillary counterdiffusion crystal growth in microgravity was successfully used to grow quality crystals of several MnSOD variants for neutron-diffraction-based mechanistic studies.

Since the process of crystallization decreases the entropy of a local system, the enthalpy must be sufficiently decreased by crystallization in order for the process to be spontaneous. This is achieved by supersaturating the protein in solution^16^. Once nucleation occurs, a crystal will grow until the local protein concentration dips below the solubility limit. For crystals to grow, a mass transport system must be established in which protein molecules move from the bulk solution to the interface between the crystal and the solution where they can be incorporated, thus maintaining supersaturation. When convection currents occur in the crystallization experiment (i.e., from vapor diffusion or the effects of gravity), a heterogeneous environment for crystal growth is created, leading to high mosaicity in large crystals^14^. Large crystal growth experiments in microgravity aim to reduce the effect of convection currents on the system.

Microgravity growth conditions and capillary-based counterdiffusion benefit large crystal growth by minimizing convective forces as determined by Grashof’s Number. Grashof’s Number (Gr_N_) is a dimensionless number that identifies the importance of convective forces for mass transport in a given system. It is described by Eq. (1):

1 $${{Gr}}_{N}=\,{L}^{3}{\beta }^{* }({C}_{c}-{C}_{s})g{v}^{-2}$$

where *L* is the length of the reaction vessel (cm), β^*^ is the solutal expansivity, which is the rate of change in density as a function of concentration (cm^3^/mg), *C*_*c*_ is the concentration of protein at the crystal’s surface, *C*_*s*_ is the concentration of protein in the solution, *g* the acceleration due to gravity (9.81 m/s^2^ on Earth), and *v* is the kinematic viscosity, which is the ratio of the fluid’s viscosity to its density^17^. Mass transport in systems with small Grashof numbers is less influenced by convection. Thus, crystallographers intending to decrease the mosaicity of large crystals seek to decrease the Grashof number of their crystallization system by either: (1) reducing *L* by crystallizing in vessels with restricted geometry, (2) increasing the viscosity of the solution, or (3) decreasing the gravity experienced by the system^14^. Capillary-based counterdiffusion experiments restrict *L* by growing crystals in long but thin capillary tubes, as well as occasionally by increasing the viscosity via the addition of carbohydrates or polymers such as polyethylene glycols. In addition, counterdiffusion experiments have been carried out on the international space station (ISS) to reduce acceleration by gravity to negligible amounts (~1 × 10^−6 ^m/s^2^)^18^, as was done in the present study.

**Fig. 2:**
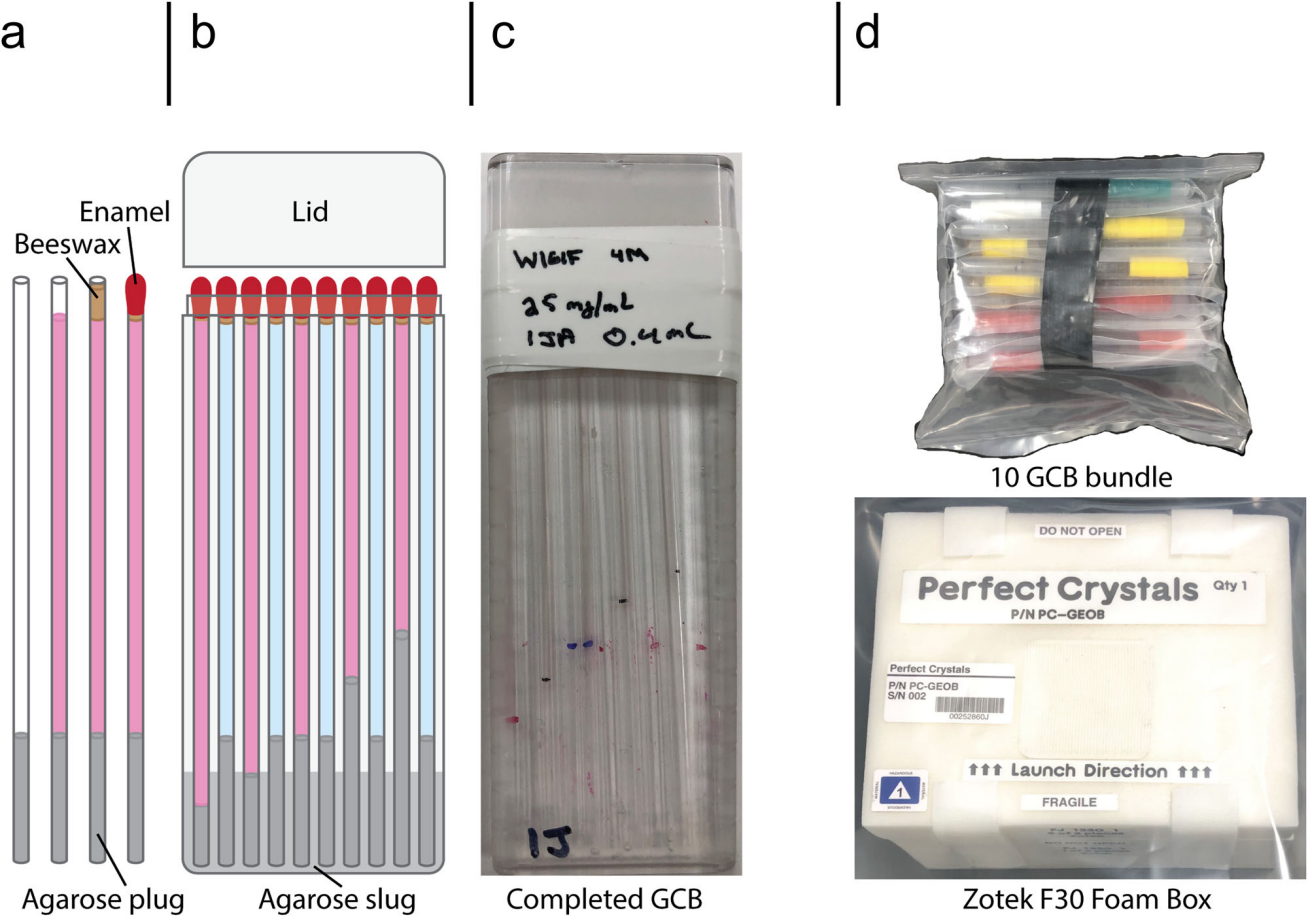
Schematic experiment preparation. **a** A quartz capillary is cut to 9 cm in length, and the desired length of molten agarose is loaded. Reservoir capillaries (depicted as blue) are loaded with 2 cm of agarose, and protein capillaries (pink) have varied agarose plug lengths. Concentrated MnSOD solution is pipetted into the capillary from the top that is sealed with molten beeswax. To ensure the capillary is watertight, enamel (fingernail polish) is applied over the end with beeswax. A volume of molten agarose (slug) is added to the GCB, and capillaries are loaded into the GCB (outer dimensions: 10-cm long, 3.5-cm wide, and 0.7-cm deep) as the agarose cools. **b** The GCB lid is filled with vacuum grease and attached to the GCB. The lid is taped on and labeled with the GCB number, initials of the preparer, and contents. **c** GCBs are individually heat-sealed into a watertight polypropylene bag before sets of 10 are taped together as a bundle and heat-sealed in a second watertight polypropylene bag. **d** A completed 10 GCB bundle is then loaded into a Zotek F30 foam box for transport to ISS. Foam boxes were given labels SN001-SN004.

**Fig. 3:**
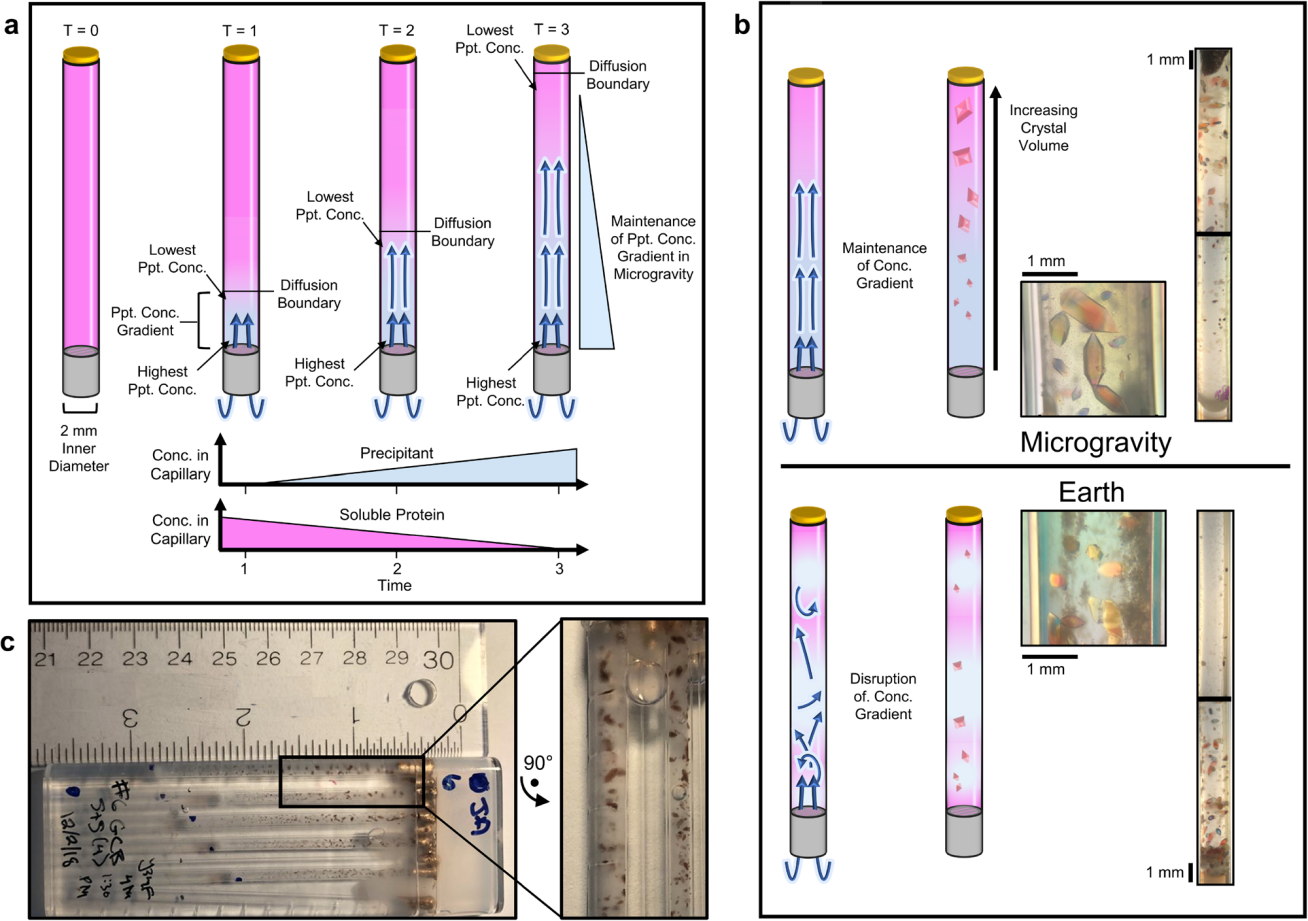
Counterdiffusion crystal growth in microgravity leads to larger and more perfect crystals compared to counterdiffusion growth on Earth. **a** In microgravity, a lack of convective motions leads to the maintenance of a precipitant concentration gradient from tempered diffusion of the precipitant (blue) into the protein solution (pink). **b** Maintenance of the concentration gradient in microgravity results in crystals of increasing size as the distance from the agarose plug (gray) increases. This gradient can be seen after return to Earth. In contrast, Earth’s gravity causes convection that disrupts the concentration gradient and sets up a continuous supply of soluble protein. **c** Representative images of large-volume crystal growth in microgravity.

Any protein’s solubility curve is dependent on both the concentration of the protein and the concentration of the precipitating agent or agents being used to induce crystallization. Counterdiffusion techniques achieve supersaturation by increasing the concentration of precipitating agent via diffusion alone. A capillary is filled with protein in solution and then sealed on one end (Fig. 2). The open end of the capillary is placed in contact with a reservoir containing the precipitating agent at a high concentration, which then diffuses into the protein solution spontaneously until equilibrium is reached. Because each cross-sectional slice of the capillary will therefore experience precipitating agent concentrations between zero and the final equilibrium concentration, the capillary contains a spatial and temporal gradient, allowing many crystallization conditions to be sampled (Fig. 3a). Conditions with high precipitant concentration yield many nucleation sites and ultimately many crystals, which are often sedimented together or twinned. Areas of lower precipitant allow for single nucleation sites to form and crystals to grow in a slow-but-steady manner that ultimately yields large and perfect crystals (Fig. 3b, c). Convection currents would disturb this diffusion gradient on Earth, allowing for mass transport of soluble protein into areas where many crystals are growing. This results in Ostwald ripening and ultimately yields many crystals clumped together near the initial diffusion front^14^. Microgravity conditions minimize these convection currents, so areas with many nucleation events do not yield large crystals since the soluble protein in the region is quickly depleted and not replaced (Fig. 3b). Often, a gel or dialysis membrane is placed between the protein solution and the reservoir to prevent the protein from diffusing out of the capillary. If a gel is used, it can also function as a time delay for crystallization since the precipitating agent must first diffuse through the gel before reaching the protein. The length and the composition of the gel can be varied to alter the amount of delay (Fig. 2), depending on what is needed. This is especially useful for experiments that need to be prepared on Earth but need to delay crystallization until a stable microgravity environment can be achieved.

**Fig. 4:**
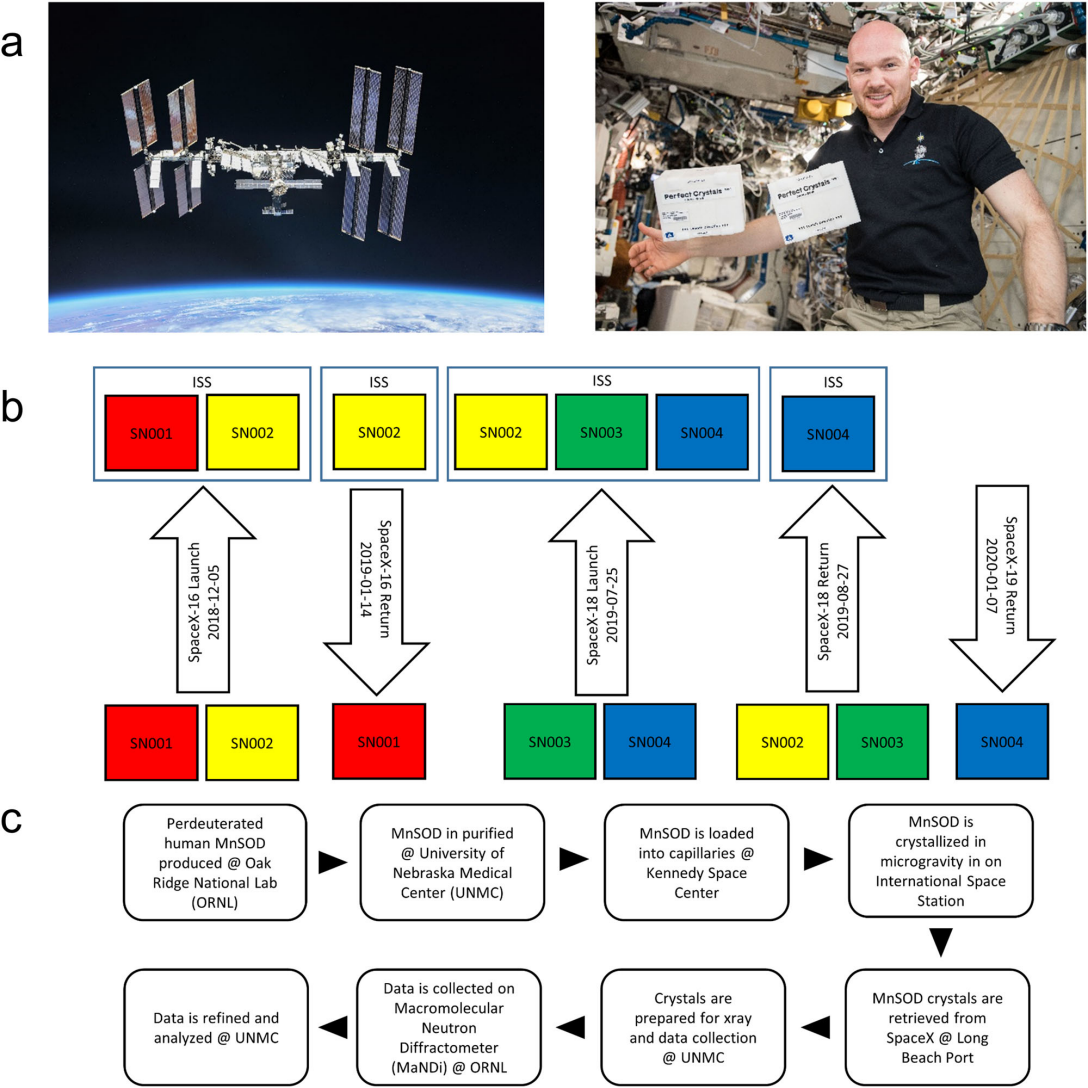
Workflow and flights. **a** Dr. Alexander Gerst is pictured with SN001 and SN002 aboard ISS. **b** Schematic representation of the spaceflight timeline. EXPRESS racks were the ISS stowage location for all boxes. **c** Workflow diagram shows the path that protein took from expression to data set. Dr. Gerst gave permission for the use of his photo.

The present study utilized microgravity-assisted capillary counterdiffusion to grow large, perfect crystals of perdeuterated MnSOD (D-MnSOD) for use in neutron-diffraction studies. Crystallization experiments were flown to ISS for wild-type and four well-characterized point mutants of human MnSOD (Fig. 4). The point mutants of MnSOD will enhance the study of its enzymatic mechanism as follows. The Trp161Phe variant forces the enzyme to commit to the product-inhibited pathway approximately 99% of the time without disrupting the hydrogen bonding network that is the object of the structural studies^19–21^. Tyr34 is the closest titratable residue to the active site Mn, making Tyr34Phe a valuable mutant for studying the active site hydrogen bond network by disrupting its native behavior^22^. Gln143 acts as a gatekeeper to the Mn^3+^ redox state, and mutation of this residue to asparagine (Gln143Asn) results in a variant that cannot perform catalysis. This variant is useful for studying the Mn^2+^ redox state, where it remains indefinitely after the oxidation of superoxide to molecular oxygen^23,24^. The next closest titratable residue to the active site Mn is His30. The His30Gln variant maintains an active hydrogen bond network while His30Asn disrupts it^25^. The NASA mission *Perfect Crystals* successfully grew large-volume, perdeuterated crystals of all these MnSOD variants. Neutron-diffraction data were collected to high resolution for future all-atom structural studies.

## Results and discussion

### Purification

In total, 2.8 g of perdeuterated MnSOD protein was purified. The purification yields for WT MnSOD and variants reach up to 21.6 mg of protein per 1 g of cell pellet though this was dependent on the variant, the amount of D_8_-glycerol used during expression and underlying differences between each of the eight runs of recombinant expression and cell harvesting (Supplementary Table 4). The lower yields of the His30 variants are attributed to the destabilization of the mutation at position 30^25,26^. Nonetheless, we demonstrate the plausibility of expressing and purifying perdeuterated recombinant proteins in the quantities necessary for large-scale crystallization experiments. Determining buffer conditions that improve and ensure long-term MnSOD solubility at the concentrations needed for concentration was an important parameter for this experiment. Some precipitation was observed visually in GCBs from boxes SN001 and SN002, but this was not an issue for the protein used in boxes SN003 and SN004 because the final buffer was changed from an MES pH 6.5 buffer system to 50 mM HEPES pH 8.2 after purification and before concentration. Ultimately these experiments demonstrate that concentrated protein can retain its stability without degrading on the timescales required for microgravity crystallization experiments conducted entirely at ambient temperatures.

### Crystallization

Large crystals of each variant were obtained. Once GCBs are at equilibrium conditions, crystals remain stable as long as they are sealed in their capillaries. This means that crystals can be stored for future data collection even if access to neutron beamlines is scarce, as was the case in 2020–2021 during the beginning of the SARS-CoV-2 pandemic. In total, 400 capillaries were flown, 204 of which were filled with protein, and of these 139 of these protein-filled capillaries yielded crystals, with 68 of those having crystallized in microgravity and 71 yielding crystals only after their return to Earth (Supplementary Table 2).

The agarose used to delay the diffusion of precipitant into the protein solution succeeded in delaying the growth of crystals by at least 33 days, even for capillaries with 2-cm agarose plugs in GCBs with 0.4 mL agarose slugs (Supplementary Table 5). It is possible nucleation conditions were reached in microgravity, and growth occurred on Earth. This allows for experiments to be loaded well in advance of launch without needing to unstow and prepare the experiments again in the event of weather-related launch delays, which occurred for SpX-18. This finding negates the necessity for the late-load requirement in future experiments. Microgravity crystallization occurred in boxes SN002 and SN004 but not in boxes SN001 or SN003, indicating that an adequate amount of time for the precipitating agent to reach growth conditions via diffusion in microgravity is between 40 and 164 days (Supplementary Table 1).

Precipitating agent concentration was an important parameter to consider during the design phase of this experiment (Supplementary Table 6). If too much precipitating agent was added, protein could precipitate before crystallization occurred. If too little precipitating agent was added, supersaturation would not be reached, and thus crystallization would not occur. The precipitating agent concentration required for crystallization is also dependent on the protein concentration. As precipitant diffuses through the agarose and into the protein, it is diluted until equilibrium is reached. The final equilibrium concentration of the precipitating agent in each GCB is between 60 and 75% of its initial concentration, depending on the GCB layout used. The precipitant concentrations tested in these experiments were 3 M and 4 M potassium phosphate buffer. The protein concentrations tested were 15 mg mL^−1^ and 25 mg mL^−1^. It was found that GCBs with a combination of 3 M precipitant and 15 mg mL^−1^ did not yield crystals, but all other combinations did yield crystals in at least one GCB (Supplementary Table 2). Pictures of all GCBs were deposited in the NASA Physical Sciences Informatics (PSI) Database.

This crystallization technique can be easily adapted for practically any protein that is stable over time after purification with known crystallization conditions, barring precipitating agents that will not diffuse through agarose or are deemed unsafe for spaceflight by NASA. The hardware components of these experiments were chosen and configured specifically to ensure that no safety hazards were created for the crew of ISS. GCBs are made of injection-molded polystyrene and are non-shattering. Heat-sealed polypropylene bags remove the hazard of liquid or gas leakage during spaceflight, and the Zotek F30 Foam boxes are fire-resistant. The safety testing and verification procedure for ISS payloads is a nontrivial process but would not need to be repeated in such depth for future experiments utilizing this hardware, allowing for greatly expedited payload development.

### Neutron data collection

High-resolution neutron-diffraction data were collected and processed to 2.28 Å resolution from a ~ 0.4 mm^3^ microgravity-grown, perdeuterated Tyr34Phe MnSOD crystal that was redox treated to the Mn^3+^ oxidation state (Supplementary Table 3). Perdeuteration alone is not enough for sufficient diffraction resolution to reveal hydrogen positions, seen at <2.5 Å. The size of the crystal was the most significant sample attribute for collecting higher-resolution data as it compensates for the lower flux of neutron beamlines. The fluxes of neutron beamlines vary between 10^6^ and 10^8^ neutrons cm^−2^ s^−1^ while synchrotron light sources harbor fluxes of greater than 10^20^ photons cm^−2^ s^−1^ ^7^. This results in diffraction intensities that are many orders of magnitudes weaker than synchrotron X-ray beams. Large unit-cell dimensions further limit diffraction due to the lesser amount of repeating units contributing towards constructive interference compared to samples of the same size but with smaller unit-cell dimensions^6^. Of note is that the unit-cell volume of *P*6_1_22 MnSOD crystals is the largest that has been studied with neutron diffraction to sufficient resolution where hydrogen positions can be discerned^7^. Here, we show the multiple layers of challenges hindering diffraction intensity are capable of being overcome by obtaining large-volume crystals grown in microgravity.

While the large unit-cell volume of the *P*6_1_22 MnSOD crystals was disadvantageous for neutron-diffraction signal size, a significant benefit was the high symmetry of the *P*6_1_22 space group. Neutron data sets often have lower completeness and multiplicity compared to X-ray counterparts due to frames of data requiring long exposures (>15 h) and limited beamtime^6^. It is noteworthy that the beamtime awarded is sufficient for the collection of data from only one crystal. Crystals for neutron data collection, unit gravity wild type, and microgravity Y34F were preselected by the quality of their X-ray diffraction using brief exposure to X-rays using our home source. The lesser amount of data becomes problematic during all-atom structure refinement, where the number of parameters to be refined are significantly greater than usual because of the deuterium/hydrogen atoms. Joint X-ray and neutron refinement^27^ serves to increase the data-to-parameter ratio though must be given careful consideration due to the known perturbations that X-rays have on solvent structure and metal redox states which are not present with neutrons^6,28^. For the present study, the symmetry of the *P*6_1_22 space group permitted a neutron data set of high completeness and multiplicity (Supplementary Table 3) to be collected from 11 frames of data over 220 h, whereas crystal forms of lower symmetry would not be able to obtain the same quality of data. Ultimately, growing large crystals in microgravity that have challenging unit-cell dimensions allowed the acquisition of high-quality neutron data that can be refined without X-ray data. This avoids including data that contains perturbations of solvent structure and redox state that are critical for deciphering the all-atom workings of MnSOD.

### Earth versus microgravity neutron results

**Fig. 5:**
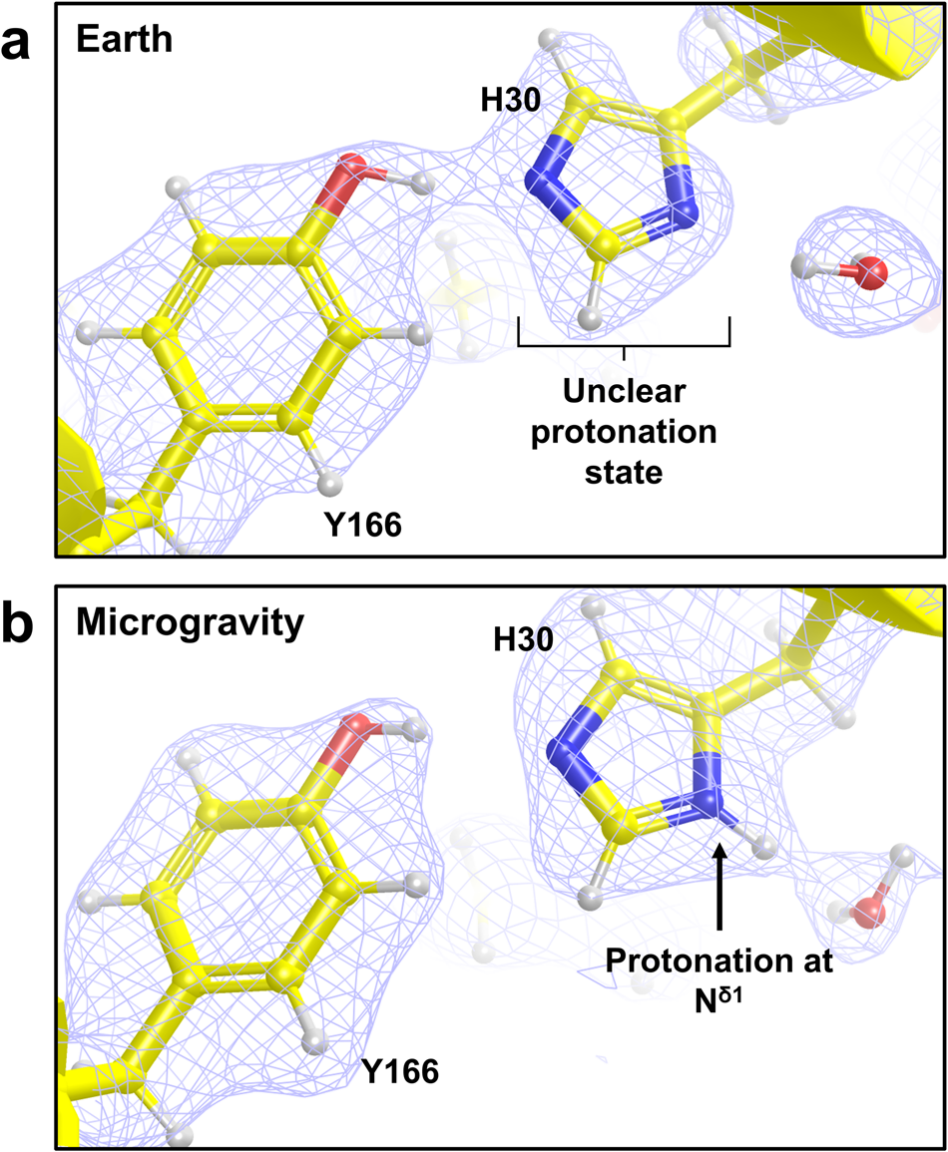
Comparison of neutron data quality at active site residues Tyr166 and His30 between Earth- and microgravity-grown perdeuterated MnSOD crystals. **a** Neutron structure from Earth-grown wild-type Mn^3+^SOD crystals with light blue 2|*F*_*o*_| − |*F*_*c*_| neutron scattering length density displayed at 1.0σ. **b** Preliminary neutron structure from microgravity-grown Tyr34Phe Mn^3+^SOD crystals with light blue nuclear 2|*F*_*o*_| − |*F*_*c*_| density displayed at 1.0σ. Both data sets were collected at room temperature and density maps are calculated at 2.28 Å resolution.

To investigate the effect of microgravity-crystal growth on the quality of neutron-diffraction data that could be obtained, 2|*F*_*o*_| − |*F*_*c*_| nuclear scattering length density maps at 1.0σ were compared at active site residues Tyr166 and His30 between neutron structures of earth-grown wild-type Mn^3+^SOD that we previously published^3^ and preliminary neutron structures of microgravity-grown Tyr34Phe Mn^3+^SOD (Fig. 5). The earth-grown neutron crystallographic data demonstrated discontinuous 2|*F*_*o*_| − |*F*_*c*_| nuclear scattering length density at His30 and its protonation state was not able to be determined (Fig. 5a). At residue Tyr166, the density of its aromatic ring is uneven. In contrast, the density at His30 from the microgravity-grown crystallographic data was continuous and more interpretable to the extent that its protonation state and hydrogen bonding with solvent was capable of being determined (Fig. 5b). For residue Tyr166, the density is even, and contours appropriately along atoms of its aromatic ring. These residues have been shown to become differentially protonated during the MnSOD catalytic cycle^3^, and knowing their protonation states is integral for deciphering mechanistic details. Importantly, the improvements in data quality mediated by microgravity-crystal growth are significant for enzymes that rely on proton transfers to facilitate catalytic activity. In terms of the enzyme mechanism, this is a significant difference in data quality as the placement of a single proton changes the net active site charge and the likelihood of an electron transfer event. In general NPC enabled by microgravity-crystal growth could benefit any protein structure problem where the precise location of protons is important to understand function.

## Methods

### Perdeuterated adaptation and expression

The pCOLADuet-1 expression vector containing full-length MnSOD cDNA optimized for *Escherichia coli* codons was transformed into BL21(DE3) cells and grown in Terrific Broth. Cell strain fidelity was maintained with 30 µg ml^−1^ kanamycin for the initial growth of cells and adaptation. Terrific Broth-grown cells were subcultured into H_2_O minimal media in preparation for deuterium adaptation which consisted of subculturing H_2_O minimal media-adapted cells into increasing concentrations of D_2_O minimal media using D_8_-glycerol as the carbon source^2,29^. For large-scale expression, 1.5 L of D_2_O minimal media within a 2.5 L sterilized bioreactor vessel was inoculated to an initial OD_600_ of 0.1–0.2, and 100 µg ml^−1^ kanamycin was used to ensure cell strain fidelity was maintained. Growth occurred at 37 °C and NaOD was added on demand to maintain a pD value > 7.3 (pD = measured pH + 0.4). Once the D_8_-glycerol was depleted from the media, indicated by a dissolved oxygen spike, a solution of 10% (w/v) D_8_-glycerol and 0.2% MgSO_4_ dissolved in D_2_O was fed into the bioreactor at 8 mL h^−1^. After ~24 h, the cells grew to an OD_600_ of 8–10 and were induced with isopropyl β-D-1-thiogalactopyranoside (IPTG) at a final concentration of 1 mM. Induction coincided with the addition of 1.4 g L^−1^ MnCl_2_ for metal incorporation. Induction lasted approximately 24 h, and the final OD_600_ measured varied between 15 and 20. Cells were harvested by centrifugation at 6000×*g*, and wet weights varied between 40 g and 60 g per batch.

### Purification

Cells were re-suspended in a solution consisting of 5 mM MnCl_2_ and 5 mM 3-(N-morpholino)propanesulfonic acid (MOPS), pH 7.8, then lysed using an Emulsiflex-C3. Cell debris was removed by centrifugation prior to heat treatment of clarified lysate at 55 °C for one hour. Precipitated proteins were removed by centrifugation while the recombinant protein remained soluble. Soluble protein was diluted threefold with 25 mM 2-(N-morpholino) ethanesulfonic acid (MES), pH 5.5, applied to a carboxymethyl (CM) sepharose (GE Healthcare) column, and eluted with a NaCl and increasing pH gradient. Eluted fractions were buffer exchanged to 50 mM 4-(2-hydroxyethyl)−1-piperazineethanesulfonic acid (HEPES) pH 8.2 and concentrated using Vivaspin 10,000 Da molecular weight cut-off spin concentrators (Sartorius) to 25 mg mL^−1^, as measured by NanoDrop ONE C (Thermo Scientific) using an extinction coefficient of 40.50 L mol^−1^ cm^−1^ at 280 nm^30^. Wild-type, Trp161Phe, Tyr34Phe, His30Asn, and His30Gln were all expressed and purified for this study, with no difference in protocol. Gln143Asn was not attempted.

### Crystallization

Granada Crystallization Boxes (GCBs) (originally made by Triana, donated by colleagues around the world, see acknowledgments) were used as the reservoir vessels for capillary counterdiffusion of perdeuterated MnSOD (Fig. 2). For each GCB, ten fused quartz capillary tubes (VitroCom) inner diameters of 2.0 mm and outer diameters of 2.4 mm were trimmed to 90 mm in length and inspected for cracks. Five of the ten capillaries that were to be filled with precipitating agent were plugged with 30 mm of molten 2% (*w*/*w*) agarose and set to cool. The remainder of the capillaries that were to be filled with protein instead had plug lengths of 20, 25, 30, 35, and 40 mm. After cooling, either protein or precipitating agent was pipetted into the capillaries with hand-fabricated pipette tips, which consisted of standard disposable micropipette tips affixed to bell-top, thin-walled glass capillaries with beeswax. Protein solution was either 15 mg mL^−1^ or 25 mg mL^−1^ perdeuterated MnSOD. Capillaries were then sealed with molten beeswax and then painted with fast-drying enamel fingernail polish. Once the polish dried, either 1 mL or 0.4 mL of molten 2% w/w agarose was added to the bottom of the GCB, and capillaries were plunged plug-first into the molten agarose, with caution to avoid air bubbles. Once the agarose had solidified, the remaining space in the GCB was filled with a precipitating agent. The precipitating agent used was either 3 M or 4 M potassium phosphate buffer, pH 7.8, which was made by combining dibasic potassium phosphate and monobasic potassium phosphate at a ratio of 91:9, respectively. Each GCB lid was filled with vacuum grease before being attached to the GCB body by electrical tape. Each GCB was individually heat-sealed in a polypropylene bag. Ten bagged GCBs were then bundled together with tape and heat-sealed as a unit into another polypropylene bag before being placed into a Zotek F30 foam box supplied by the Cargo Mission Contract provider, Leidos. In total, 40 GCBs were prepared, split between four foam boxes (Fig. 4 and Supplementary Tables 1 and 2). All experiments were prepared at the Space Station Processing Facility (SSPF) at Kennedy Space Center. The boxes were late loaded onto SpaceX CRS Dragon capsules in ambient stowage. All boxes were stowed in Lab1O2_L2 in the Destiny Module of ISS. (In Fig. 4, Dr. Gerst gave his permission for the use of his photo.) None of the boxes were opened, and no crew interaction was required. The boxes were only moved when the EXPRESS racks were involved in unscheduled operations, which occurred several times during the experiment. It was requested that the boxes be moved with acceleration loads vectored only perpendicular to the length of the capillaries, which was indicated on the outside of each box with a label marked “Return Vector” and an arrow. On return, boxes were stowed with the return vector facing Earth as indicated. Crystals were cataloged and photographed upon return to Borgstahl lab in Omaha.

### Extracting samples from GCBs

Our original plan was to directly mount the perdeuterated microgravity crystals in the quartz capillaries they grew in. The capillaries were too crowded with crystals to accomplish this. So, single crystals were extracted from the GCBs for data collection, and this was performed by first removing the GCB lid and carefully pulling out the capillary containing the desired crystals with tweezers. Prior to opening the capillary, 1 mL of the precipitating agent from the GCB was pipetted to a well of a nine-well glass plate to serve as an area for crystals to be manipulated. The capillary was then broken at both ends with a scoring stone, while the agarose end was pointed down and placed directly above the well containing precipitating agent. The contents of the capillary were transferred into the well by gently pipetting precipitating agent into the capillary end opposite the well.

### Crystal mounting

Mounting samples for neutron diffraction used thick-walled quartz capillaries (2.0-mm inner diameter × 2.4-mm outer diameter) that were preferred for transparency to neutrons and durability. Single crystals were carefully drawn into the capillaries with tubing attached to a Captrol III aspirator (Drummond Scientific) that used a knob to aspirate volumes of 20 µL volumes per turn. Once a crystal was inside, the innards of the capillary were dried with paper wicks, but a conservative amount of liquor was left on the crystal to prevent drying.

### Deuterium exchange and redox manipulation

Initial deuterium exchange of a crystal occurred within the capillary mount by placing 10 µL slugs of deuterated solution on flanking sides of the crystal and sealing the capillary at both ends with beeswax. The deuterated solution consisted of 4 M KD_2_PO_4_ and K_2_DPO_4_ at pH 7.4 (adjusted by varying ratios of monobasic and dibasic forms), which is the equivalent pD of 7.8. The pD value is calculated by adding 0.4 to the measured pH reading. Deuterated solutions were replaced with new ones daily over the course of the week to replace the hydrogen within the capillary. After the week of the initial deuterium exchange, the crystal was soaked in deuterated solution supplemented with 6.4 mM potassium permanganate (KMnO_4_) within the capillary to achieve the Mn^3+^SOD oxidation state (as opposed to a mix of Mn^3+^ and Mn^2+^). Without the initial exchange, crystals were vulnerable to cracking during soaking. After soaking for three days, the capillary was dried with the exception of the small amount of liquor left on the crystal, and KMnO_4_-supplemented deuterated solutions were placed on either side of the crystal. After the final sealing with beeswax, capillary ends were coated with fingernail polish to serve as an additional layer of sealant.

### Neutron data collection and processing

Laue neutron diffraction using the MaNDi instrument at the Spallation Neutron Source was used to collect data, and diffracted neutrons were wavelength resolved by time-of-flight^31,32^. Data were collected on a 0.40 mm^3^ perdeuterated KMnO_4_-treated Tyr34Phe MnSOD crystal to 2.28 Å resolution using all neutrons with wavelengths between 2 and 4 Å (Supplementary Table 3). Each frame of the diffracted data was from the sample held at a stationary position and was rotated along the Φ axis for successive frames at 20° increments. For data reduction, data were integrated using the three-dimensional profile fitting algorithm^33^ from the MANTID software package^34^, while scaling and wavelength-normalization used the Lauenorm program from the LAUGEN software package^35^.

### Reporting summary

Further information on research design is available in the Nature Research Reporting Summary linked to this article.

## Supplementary information

Supplementary Information

Reporting Summary

## Data Availability

The structural data sets generated and analyzed during the current study are available from the corresponding author on reasonable request and will be submitted to the Protein Data Bank once the refinement, analysis, and final publication of these results are finished. The raw data (photos of crystal setups) is available through NASA’s Physical Science Informatics (PSI) Database under the CVB experiment (https://www.nasa.gov/PSI).
