# Supplementary material for: Perfect Crystals: microgravity capillary counterdiffusion crystallization of human manganese superoxide dismutase for neutron crystallography
Source: NPJ Microgravity. 2023 Jun 3;9:39. doi: 10.1038/s41526-023-00288-x (PMC10238240; doi:10.1038/s41526-023-00288-x)
Supplement: Supplementary file 1 — Supplementary Information [file 41526_2023_288_MOESM1_ESM.pdf]

SUPPLEMENTARY INFORMATION

Perfect Crystals: Microgravity capillary counterdiffusion crystallization of human manganese superoxide dismutase for neutron crystallography

William E. Lutz[1], Jahaun Azadmanesh[1], Jeffrey J. Lovelace[1], Carol Kolar[1], Leighton Coates[3], Kevin L. Weiss[4], and Gloria E. O. Borgstahl[1,2*]

[1] Eppley Institute for Cancer and Allied Diseases, 986805 Nebraska Medical Center, Omaha, NE 68198-6805, USA
[2] Department of Biochemistry and Molecular Biology, 985870 Nebraska Medical Center, Omaha, NE 68198-5870, USA
[3] Second Target Station, Oak Ridge National Laboratory, 1 Bethel Valley Road, Oak Ridge, TN 37831, USA
[4] Neutron Scattering Division, Oak Ridge National Laboratory, 1 Bethel Valley Road, Oak Ridge, TN 37831, USA
*gborgstahl@unmc.edu

Supplementary Table 1: Duration of exposures to microgravity.

Box	Launch Date	Return Date	Time in Microgravity
SN001	2018-12-05	2019-01-14	40 days
SN002	2018-12-05	2019-08-27	263 days
SN003	2019-07-25	2019-08-27	33 days
SN004	2019-07-25	2020-01-07	164 days

Supplementary Table 2. Box Contents

Box SN001						
**GCB #**	**Variant**	**[Protein]**	**[Reservoir]**	**Crystals**	**Gravity**	**Layout**
GCB 1	WT	25 mg/mL	4M	Y*	Earth*	A
GCB 2	WT	25 mg/mL	4M	Y	Earth	A
GCB 3	WT	25 mg/mL	3M	N	Earth	A
GCB 4	WT	25 mg/mL	4M	Y	Earth	C
GCB 5	Y34F	25 mg/mL	4M	Y	Earth	A
GCB 6	Y34F	25 mg/mL	4M	Y	Earth	A
GCB 7	Y34F	25 mg/mL	3M	Y	Earth	D
GCB 8	Y34F	25 mg/mL	3M	Y	Earth	D
GCB 9	H30N	25 mg/mL	4M	Y	Earth	A
GCB 10	H30Q	25 mg/mL	4M	Y	Earth	B
**Box SN002**						
**GCB #**	**Variant**	**[Protein]**	**[Reservoir]**	**Crystals**	**Gravity**	**Layout**
GCB 1	WT	25 mg/mL	4M	Y	Microgravity	A
GCB 2	WT	25 mg/mL	4M	Y	Microgravity	A
GCB 3	WT	25 mg/mL	3M	N	Microgravity	A
GCB 4	WT	25 mg/mL	4M	Y	Microgravity	C
GCB 5	Y34F	25 mg/mL	4M	Y	Microgravity	A
GCB 6	Y34F	25 mg/mL	4M	Y	Microgravity	A
GCB 7	Y34F	25 mg/mL	3M	N	Microgravity	A
GCB 8	Y34F	25 mg/mL	3M	Y	Microgravity	D
GCB 9	H30N	25 mg/mL	4M	Y	Microgravity	A
GCB 10	H30Q	25 mg/mL	4M	Y	Microgravity	B
**Box SN003**						
**GCB #**	**Variant**	**[Protein]**	**[Reservoir]**	**Crystals**	**Gravity**	**Layout**
GCB 1	W161F	25 mg/mL	4M	Y	Earth	E
GCB 2	W161F	25 mg/mL	3M	N	Earth	E
GCB 3	W161F	15 mg/mL	4M	Y	Earth	E
GCB 4	W161F	15 mg/mL	3M	N	Earth	E
GCB 5	W161F	15 mg/mL	4M	N	Earth	A
GCB 6	W161F	15 mg/mL	3M	N	Earth	A
GCB 7	WT	15 mg/mL	4M	Y	Earth	E
GCB 8	WT	15 mg/mL	3M	N	Earth	E
GCB 9	Y34F	15 mg/mL	4M	Y	Earth	E
GCB 10	Y34F	15 mg/mL	3M	N	Earth	E
**Box SN004**						
**GCB #**	**Variant**	**[Protein]**	**[Reservoir]**	**Crystals**	**Gravity**	**Layout**

GCB 1	W161F	25 mg/mL	4M	Y	Microgravity	E
GCB 2	W161F	25 mg/mL	3M	Y	Microgravity	E
GCB 3	W161F	15 mg/mL	4M	Y	Microgravity	E
GCB 4	W161F	15 mg/mL	3M	N	Microgravity	E
GCB 5	W161F	15 mg/mL	4M	Y	Microgravity	A
GCB 6	W161F	15 mg/mL	3M	N	Microgravity	A
GCB 7	WT	15 mg/mL	4M	Y	Microgravity	E
GCB 8	WT	15 mg/mL	3M	N	Microgravity	E
GCB 9	Y34F	15 mg/mL	4M	Y	Microgravity	E
GCB 10	Y34F	15 mg/mL	3M	N	Microgravity	E

*Y=Yes; N=No, Earth = crystal growth occurred on earth in unit gravity, Microgravity = crystal growth occurred in the microgravity environment on ISS.

Supplementary Table 3. Neutron data collection statistics.

	Y34F Mn^{3+}SOD
Diffraction Source	MaNDi
Wavelengths (Å)	2-4
Temperature (K)	296
Detectors	40 SNS Anger Cameras
Crystal-to-detector distance (mm)	450
Rotation along Φ between images (°)	20
Total rotation range (°)	220
No. of images collected	11
Exposure time per image (hrs)	20
Space group	$P6_122$
a, b, c (Å)	79.3, 79.3, 240.6
α, β, γ (°)	90, 90, 120
Resolution range (Å)	14.39-2.28 (2.36-2.28)
Total No. of reflections	161187
No. of unique reflections	20703
Completeness (%)	97.78 (88.40)
Multiplicity	7.79 (6.15)
I/σ(I)	7.4 (3.7)
R_{merge}	0.246 (0.323)
R_{meas}	0.263 (0.349)
$R_{p.i.m.}$	0.090 (0.127)

Supplementary Table 4. Purification yields for deuterated wild-type MnSOD and its variants.

MnSOD type	Pellet weight (g)	Purified Protein (mg)	Protein/Pellet (mg/g)
Wild-type	120	1530	12.8
Tyr34Phe	62	276	4.5
His30Gln	35	31	0.9
His30Asn	38	94	2.5
Trp161Phe	41	887	21.6

Supplementary Table 5. GCB Layouts

GCB Layout A (1 mL agarose slug)		
**Capillary #**	**Content**	**Agarose Plug Length (cm)**
1	Protein	2
2	Reservoir	3
3	Protein	2.5
4	Reservoir	3
5	Protein	3
6	Reservoir	3
7	Protein	3.5
8	Reservoir	3
9	Protein	4
10	Reservoir	3

GCB Layout D (1 mL agarose slug)		
**Capillary #**	**Content**	**Agarose Plug Length (cm)**
1	Reservoir	2
2	Reservoir	3
3	Reservoir	2.5
4	Reservoir	3
5	Protein	3
6	Reservoir	3
7	Protein	3.5
8	Reservoir	3
9	Protein	4
10	Reservoir	3

GCB Layout C (1 mL agarose slug)		
**Capillary #**	**Content**	**Agarose Plug Length (cm)**
1	Reservoir	3
2	Protein	2.5
3	Reservoir	3
4	Protein	3
5	Reservoir	3
6	Protein	3.5
7	Reservoir	3
8	Protein	4
9	Reservoir	3
10	Protein	3

GCB Layout E (0.4 mL agarose slug)		
**Capillary #**	**Content**	**Agarose Plug Length (cm)**
1	Protein	2
2	Reservoir	3
3	Protein	2.5
4	Reservoir	3
5	Protein	3
6	Reservoir	3
7	Protein	3.5
8	Reservoir	3
9	Protein	4
10	Reservoir	3

GCB Layout C (1 mL agarose slug)		
**Capillary #**	**Content**	**Agarose Plug Length (cm)**
1	Protein	2
2	Protein	2
3	Protein	2.5
4	Protein	2.5
5	Protein	3
6	Protein	3
7	Protein	3.5
8	Protein	3.5
9	Protein	4
10	Protein	4

Supplementary Table 6. Precipitant Dilution Factors for GCB Layouts

	Volumes (mL)						
**GCB Layout**	**Quartz**	**Agarose**	**Protein**	**Reservoir**	**GCB (sans lid)**	**GCB-Quartz**	**Precipitant Dilution Factor***
Layout A	0.5	1.94	0.94	6.62	10	9.5	**1.44**
Layout B	0.5	1.97	0.91	6.62	10	9.5	**1.44**
Layout C	0.5	1.94	1.88	5.67	10	9.5	**1.67**
Layout D	0.5	1.94	0.52	7.04	10	9.5	**1.35**
Layout E	0.5	1.34	0.94	7.22	10	9.5	**1.32**

*dilution factor= (GCB-Quartz)/(Reservoir)
